# A machine learning model predicts stroke associated with blood cadmium level

**DOI:** 10.1038/s41598-024-65633-w

**Published:** 2024-06-26

**Authors:** Wenwei Zuo, Xuelian Yang

**Affiliations:** 1 School of Gongli Hospital Medical Technology, University of Shanghai for Science and Technology, No. 516, Jungong Road, Yangpu Area, Shanghai, 200093 China; 2 Department of Neurology, Shanghai Pudong New Area Gongli Hospital, No. 219 Miaopu Road, Pudong New Area, Shanghai, 200135 China

**Keywords:** Stroke, Cadmium exposure, Machine learning, NHANES, Predictive model, Interpretable, Health occupations, Neurology, Risk factors

## Abstract

Stroke is the leading cause of death and disability worldwide. Cadmium is a prevalent environmental toxicant that may contribute to cardiovascular disease, including stroke. We aimed to build an effective and interpretable machine learning (ML) model that links blood cadmium to the identification of stroke. Our data exploring the association between blood cadmium and stroke came from the National Health and Nutrition Examination Survey (NHANES, 2013–2014). In total, 2664 participants were eligible for this study. We divided these data into a training set (80%) and a test set (20%). To analyze the relationship between blood cadmium and stroke, a multivariate logistic regression analysis was performed. We constructed and tested five ML algorithms including K-nearest neighbor (KNN), decision tree (DT), logistic regression (LR), multilayer perceptron (MLP), and random forest (RF). The best-performing model was selected to identify stroke in US adults. Finally, the features were interpreted using the Shapley Additive exPlanations (SHAP) tool. In the total population, participants in the second, third, and fourth quartiles had an odds ratio of 1.32 (95% CI 0.55, 3.14), 1.65 (95% CI 0.71, 3.83), and 2.67 (95% CI 1.10, 6.49) for stroke compared with the lowest reference group for blood cadmium, respectively. This blood cadmium-based LR approach demonstrated the greatest performance in identifying stroke (area under the operator curve: 0.800, accuracy: 0.966). Employing interpretable methods, we found blood cadmium to be a notable contributor to the predictive model. We found that blood cadmium was positively correlated with stroke risk and that stroke risk from cadmium exposure could be effectively predicted by using ML modeling.

## Introduction

Stroke is the leading cause of death and disability worldwide^1^. Each year, approximately 800,000 Americans experience a new or recurrent stroke, and approximately 140,000 die from stroke^2^. However, the stroke determinants are multifaceted and complex, with hypertension, hyperlipidemia, other blood lipids, diabetes mellitus, sedentary lifestyle, and cardiac arrhythmias being recognized as major risk factors for stroke^3^. In addition, environmental pollution has attracted considerable attention in recent studies^4–7^, and cadmium is a prevalent environmental toxicant that may contribute to cardiovascular disease^8–10^, including stroke^11^. Most of these studies used traditional statistical analysis. Therefore, a novel analytical method may help to more accurately identify the relationship between stroke and cadmium exposure.

ML has been used to compare risk assessment indicators for stroke prediction based on traditional regression models. It has been found that ML can incorporate large amounts of data, aid in the identification of new risk factors, achieve superior performance, and provide feature interpretation for prediction^12^. Unlike traditional prediction models that use selected variables for computation, machine learning techniques can easily incorporate a large number of variables as all computations are performed using a computer^13^. These characteristics enable the application of machine learning techniques in the medical domain. In stroke, machine learning techniques are increasingly used in various fields, including the prediction of stroke risk factors^14–16^.

In this study, we aimed to develop ML prediction models for stroke using blood cadmium data from the NHANES 2013–2014. SHAP (Shapley Additive exPlanations) is a novel approach to interpreting ML models based on game theory, which is capable of both local and global interpretation and has been validated in other studies^17–19^. We identified five ML models that can be used for blood cadmium to identify stroke and compared the performance characteristics of the models. In addition, our study incorporates an advanced ML technique based on SHAP to determine the contribution of blood cadmium to stroke identification.

## Methods

### Study population

The data used in this study were obtained from NHANES, a nationally representative, continuous, open-ended, multi-objective survey established by the Centers for Disease Control and Prevention (CDC) to assess the health status of citizens in the U.S. This study was conducted in accordance with the Declaration of Helsinki. NHANES received approval from the Ethics Review Board of the National Center for Health Statistics (NCHS). All participants provided written informed consent. This report was based on the Strengthening the Reporting of Observational Studies in Epidemiology (STROBE) guidelines for cross-sectional studies.

**Figure 1:**
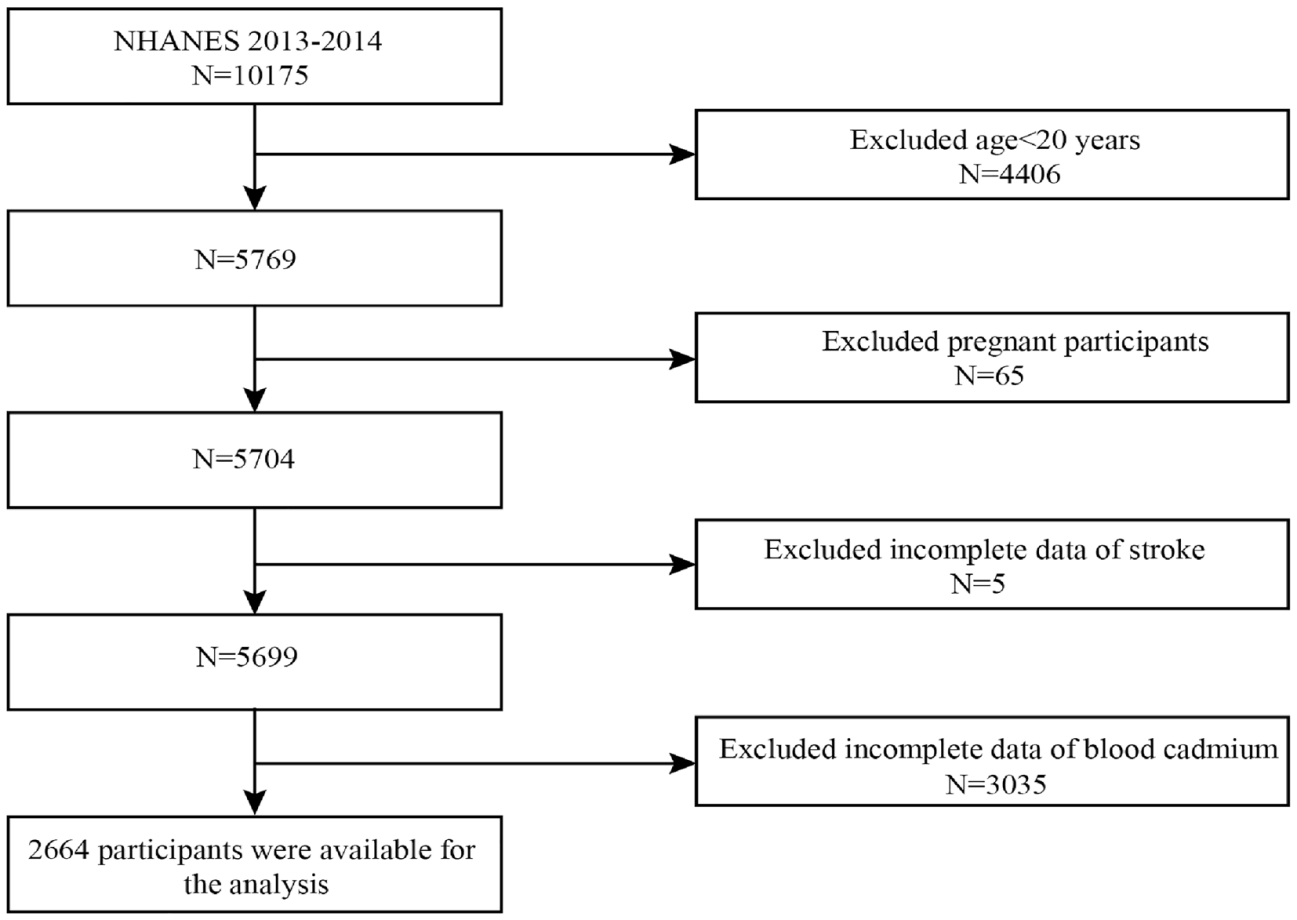
Flowchart for the selection of eligible participants from NHANES 2013–2014. 10,175 participants in the NHANES 2013–2014, we excluded 4406 participants under 20 years of age, 5 participants with missing data on stroke, and 3035 participants with missing data on blood cadmium, for a total of 2664 participants included in this study. *NHANES* National Health and Nutrition Examination Survey.

As shown in Fig. 1, the NHANES 2013–2014 included 10,175 participants. We initially screened 5769 subjects aged 20 years or older. Among these participants, pregnant women or individuals with missing blood cadmium or stroke data were excluded. Ultimately, 2664 participants were included in the final analysis.

### Data collection

Participants' demographic and socioeconomic characteristics were collected in demographic data from NHANES in the United States. Characteristics included gender, age (years), race (Mexican American, other Hispanic, non-Hispanic white, non-Hispanic black, other race), educational level (less than high school, high school or equivalent, and college or higher), and poverty-to-income ratio (PIR) (≤ 0.99, 1–3.99, ≥ 4). Body mass index (BMI) was collected in the examination data. Dietary information on total energy intake was collected through 24-h meal reviews. Information on alcohol consumption and current smoking status was collected through questionnaire data.

### Assessment of blood cadmium

Whole blood specimens were processed, stored, and shipped to the Laboratory Sciences Division, National Center for Environmental Health, and Centers for Disease Control and Prevention for analysis. Dilution of the blood during the sample preparation step before analysis was a simple dilution of 1 part sample + 1 part water + 48 parts diluent, and direct cadmium concentrations in these samples were measured using inductively coupled plasma mass spectrometry (ICP-MS)^20^. Detailed laboratory procedures and blood sample collection and processing methods are available at the following link: https://wwwn.cdc.gov/nchs/data/nhanes/2013-2014/labmethods/PbCd_H_MET.pdf. The blood cadmium measurement was divided by the square root of 2 if it was below the lower limit of detection. The NHANES Quality Assurance and Quality Control (QA/QC) protocols were following the provisions of the Clinical Laboratory Improvement Act of 1988. All test results complied with the Laboratory Sciences Division quality control standards and the quality assurance performance standards for accuracy and precision. Detailed QA/QC instructions are available at: https://wwwn.cdc.gov/nchs/data/nhanes/2013-2014/manuals/2013_mec_laboratory_procedures_manual.pdf.

### Evaluation of stroke

The outcome of this study was self-reported stroke. We confirmed the occurrence of a stroke event by asking participants the following question: "Have you ever been told by a doctor or other health care professional that you have experienced a stroke?".

### Preprocessing and ML modeling strategies

Missing values for continuous variables were filled with their median, while for categorical variables, the nearest fill method was applied^21^. In the ML model setup, features were normalized using standard scaler. Categorical variables were represented using one-hot coding, which increased the dimensionality of the variable^22^.The study data was divided into 80% as a training set (n = 2131) and 20% as a test set (n = 533)^23^. We used tenfold cross-validation to evaluate the predictive performance of the model, as it is the most commonly used method in machine learning-based exploration of medical problems^24^. We used five different ML models, namely, K-nearest neighbor (KNN), decision tree (DT), logistic regression (LR), multilayer perceptron (MLP), and random forest (RF), to identify blood cadmium-induced stroke.. KNN has high accuracy, insensitivity to outliers, and no assumptions about data inputs, but the intertemporal complexity is overwhelming^21^. DT is capable of handling missing values, outliers, and noisy data. However, they are prone to overfitting and less stable^25^. Logistic regression is characterized by simplicity and high interpretability^26^. Specifically, the logistic regression model uses the following formula to predict the probability of the target class:

1 $$h\left(w\right)=w1x1+w2x2+w3x3+\dots +b,$$

where ${x}_{1}$, ${x}_{2}$, …., ${x}_{n}$ are the input features, ${w}_{1}$, ${w}_{2}$, …, ${w}_{n}$ are the corresponding weights, and $b$ is the bias term. The predicted probability is then obtained by applying the sigmoid function to the linear combination:

2 $$g({w}^{T}x) =\frac{1}{1+{e}^{-h (w)}}=\frac{1}{1+{e}^{-(w1x1+w2x2+w3x3+\dots +b)}},$$

where $g({w}^{T}x)$ represents the probability that the input features $x$ belong to the target class. MLP has strong nonlinear learning ability but is prone to overfitting^27^. RF is characterized by the automatic selection of important features and prevention of overfitting, but it is computationally expensive^28^. We used the grid search method to tune the hyperparameters. Table S1 provides the optimization parameters for each ML algorithm. The trained models were then validated on a test dataset, and the output was an evaluation metric of the model performance. The performance of the models was assessed by a variety of evaluation metrics such as receiver operating characteristic (ROC), area under the operator curve (AUC), accuracy, sensitivity/recall, specificity, negative predictive value (NPV), false positive rate (FPR), false negative rate (FNR), and F1 score.

In addition, we used SHAP to interpret and visualize the effect of predictors on stroke risk. By averaging the Shapley values for each variable across all study samples, we can rank and evaluate their importance in the predictive model. The Shapley values apply to both the categorization of variable importance and the categorization of variable effects. A summary chart combines variable importance and effects. Each point on the summary chart represents a Shapley value for a variable and a specific data point. The colors represent the values of the variables from low (blue) to high (red), and the overlapping points are identified on the y-axis. In this paper, positive Shapley values indicate a higher likelihood of positive outcomes (diagnosis of stroke), while negative values suggest the opposite. All algorithms were implemented in Python (version 3.12.0).

### Statistical analyses

In this study, blood cadmium concentrations were categorized into quartiles based on the distribution of blood cadmium concentrations in the study population (quartile 1: < 25th percentile, quartile 2: ≥ 25th-50th percentile, quartile 3: ≥ 50th-75th percentile, quartile 4: ≥ 75th percentile), with quartile 1 serving as the reference category. Normally distributed variables were described by mean ± standard deviation, and non-normally distributed variables were described by median (interquartile range). Analysis of variance (ANOVA) was used to compare differences between groups when the variable met a normal distribution, otherwise, nonparametric tests were used. Counts (percentages) were used for the description of categorical variables, and chi-square tests were used to compare the distribution of categorical variables between groups. The odds ratio (OR) and 95% confidence interval (CI) were calculated. Multivariable logistic regression models were used to assess the relationship between blood cadmium concentration and stroke prevalence. The crude model did not adjust for any potential confounders. In model 1, we adjusted for gender, age, and race. In model 2, we further adjusted for education level, PIR, BMI, energy intake, alcohol consumers, and current smokers. Statistical analyses were performed using SPSS software (version 26.0), and P < 0.05 was considered a statistically significant difference.

## Results

### Demographic characteristics

The general demographic characteristics of the study subjects, as well as the quartiles of blood cadmium concentrations, are shown in Table 1. Subjects with higher blood cadmium concentrations tended to be older and had lower BMI and energy intake compared to those with lower blood cadmium concentrations. In the higher quartiles of blood cadmium, there were significantly higher proportions of females, those with high school or equivalent education, those with lower household incomes, current smokers, and stroke patients.

**Table 1:** Baseline characteristics for the total participants, as well as stratified by quartile of blood cadmium.

Characteristic	All (N = 2664)	Q1 (≤ 1.60) (N = 682)	Q2 (1.61–2.67) (N = 685)	Q3 (2.68–5.25) (N = 637)	Q4 (≥ 5.26) (N = 660)	*P*-value
Gender, n (%)						< 0.001
Male	1289 (48.39)	417 (61.14)	332 (48.47)	249 (39.09)	291 (44.09)	
Female	1375 (51.61)	265 (38.86)	353 (51.53)	388 (60.91)	369 (55.91)	
Age^a^ (years)	49 (35, 63)	40 (28, 54)	48 (34, 65)	56 (42, 67)	52 (38, 64)	< 0.001
Race, n (%)						< 0.001
Mexican American	365 (13.71)	101 (14.81)	134 (19.56)	84 (13.19)	46 (6.97)	
Other Hispanic	238 (8.93)	77 (11.29)	60 (8.76)	57 (8.95)	44 (6.67)	
Non-Hispanic White	1162 (43.62)	330 (48.39)	285 (41.61)	257 (40.35)	290 (43.94)	
Non-Hispanic Black	515 (19.33)	123 (18.03)	122 (17.81)	116 (18.21)	154 (23.33)	
Other race	384 (14.41)	51 (7.48)	84 (12.26)	123 (19.30)	126 (19.09)	
Education level, n (%)						< 0.001
Less than high school	233 (8.75)	51 (7.48)	57 (8.32)	62 (9.73)	63 (9.55)	
High school or equivalent	949 (35.62)	227 (33.28)	214 (31.24)	189 (29.67)	319 (48.33)	
College or above	1482 (55.63)	404 (59.24)	414 (60.44)	386 (60.60)	278 (42.12)	
PIR, n (%)						< 0.001
Low	551 (20.68)	127 (18.62)	106 (15.47)	110 (17.27)	208 (31.52)	
Medium	1465 (56.00)	372 (54.55)	376 (54.89)	353 (55.42)	364 (55.15)	
High	648 (23.32)	183 (26.83)	203 (29.64)	174 (27.31)	88 (13.33)	< 0.001
BMI^a^, kg/m^2^	27.90 (24.20, 32.40)	28.80 (24.90, 34.33)	28.00 (25.00, 32.70)	28.10 (24.40, 31.95)	26.40 (22.80, 30.78)	< 0.001
Energy intake^b^, kcal/day	4061.59 ± 1507.79	4251.08 ± 1511.82	4061.50 ± 1487.80	3913.91 ± 1486.56	4008.41 ± 1588.80	
Alcohol consumer, n (%)						0.087
No	743 (27.89)	185 (27.13)	192 (28.03)	200 (31.40)	166 (25.15)	
Yes	1921 (72.11)	497 (72.87)	493 (71.97)	437 (68.60)	494 (74.85)	
Current smoker, n (%)						< 0.001
No	2134 (80.11)	673 (98.68)	644 (94.01)	550 (86.34)	267 (40.45)	
Yes	530 (19.89)	9 (1.32)	41 (5.99)	87 (13.66)	393 (59.55)	
Stroke, n (%)						< 0.001
No	2581 (96.88)	674 (98.83)	668 (97.52)	612 (96.08)	627 (95.00)	
Yes	83 (3.12)	8 (1.17)	17 (2.48)	25 (3.92)	33 (5.00)	

*BMI* body mass index, *PIR* poverty to income ratio.

^a^Values are median (interquartile range), ^b^Values are mean (standard deviation).

### Blood cadmium concentration and stroke

As shown in Table 2, in the most adjusted model, participants in quartiles 2, 3, and 4 had an OR for stroke compared with the lowest reference group for blood cadmium in the total population, with OR of 1.32 (95% CI 0.55–3.14), 1.65 (95% CI 0.71–3.83), and 2.67 (95% CI 1.10–6.49), respectively, with a significant trend (p = 0.008).

**Table 2:** The association of blood cadmium concentrations with stroke prevalence, NHANES 2013–2014.

Outcomes	Quartile blood cadmium level (μmol/L)				*P* for trend
	Q1 (≤ 1.60) (N = 682)	Q2 (1.61–2.67) (N = 685)	Q3 (2.68–5.25) (N = 637)	Q4 (≥ 5.26) (N = 660)	
OR (95% CI)					
Crude model	1.00 (ref)	2.14 (0.92, 5.00)	3.44 (1.54, 7.69)	4.43 (2.03, 9.67)	< 0.001
Model 1	1.00 (ref)	1.26 (0.53, 3.00)	1.65 (0.72, 3.78)	2.66 (1.19, 5.95)	< 0.001
Model 2	1.00 (ref)	1.32 (0.55, 3.14)	1.65 (0.71, 3.83)	2.67 (1.10, 6.49)	0.008

Crude model: did not adjust any potential confounders. Model 1: adjusted for gender, age, and race. Model 2: further adjusted education level, poverty to income ratio, body mass index, energy intake, alcohol consumer, current smoker.

### Evaluating ML model performance

**Figure 2:**
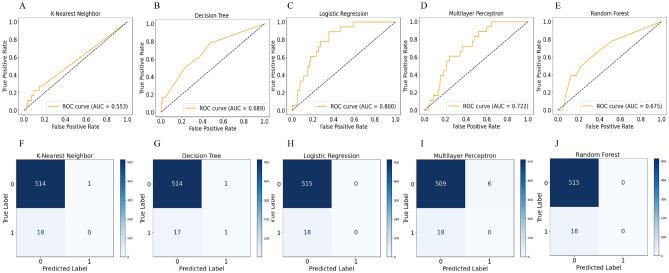
The area under the operator curve (AUC) and confusion matrix for models. Figure (**A**–**E**) depicts the ROC curves of the five models. The AUC values of the KNN (**A**), DT (**B**), LR (**C**), MLP (**D**), and RF (**E**) models in the test set are 0.553, 0.689, 0.800, 0.722, and 0.675, respectively. Figure (**F**–**J**) shows the confusion matrix for KNN (**F**), DT (**G**), LR (**H**), MLP (**I**), and RF (**J**), respectively.

For testing, we applied the trained models to the test set. The LR model demonstrated the best AUC performance (AUC: 0.800), followed by MLP (0.722), DT (0.689), RF (0.675), and KNN (0.553)models. The AUC, accuracy, sensitivity/recall, specificity, negative predictive value (NPV), false positive rate (FPR), false negative rate (FNR), and F1 score of the five ML models are shown in Table 3. The ROC and confusion matrix for all models are shown in Fig. 2.

**Table 3:** Comparison of discrimination characteristics among five ML models.

Characteristics	KNN	DT	LR	MLP	RF
AUC	0.553	0.689	0.800	0.722	0.675
Accuracy	0.964	0.966	0.966	0.954	0.966
Sensitivity/recall	0.000	0.055	0.000	0.000	0.000
Specificity	0.998	0.998	1.000	0.988	1.000
NPV	0.966	0.967	0.966	0.965	0.966
FPR	0.001	0.001	0.000	0.011	0.000
FNR	1.000	0.944	1.000	1.000	1.000
F1 score	0.490	0.545	0.490	0.490	0.490

*KNN* K-nearest neighbor, *DT* decision tree, *LR* logistic regression, *MLP* multilayer perceptron, *RF* random forest, *AUC* area under the operator curve, *NPV* negative predictive value, *FPR* false positive rate, *FNR* false negative rate.

### Interpretation

**Figure 3:**
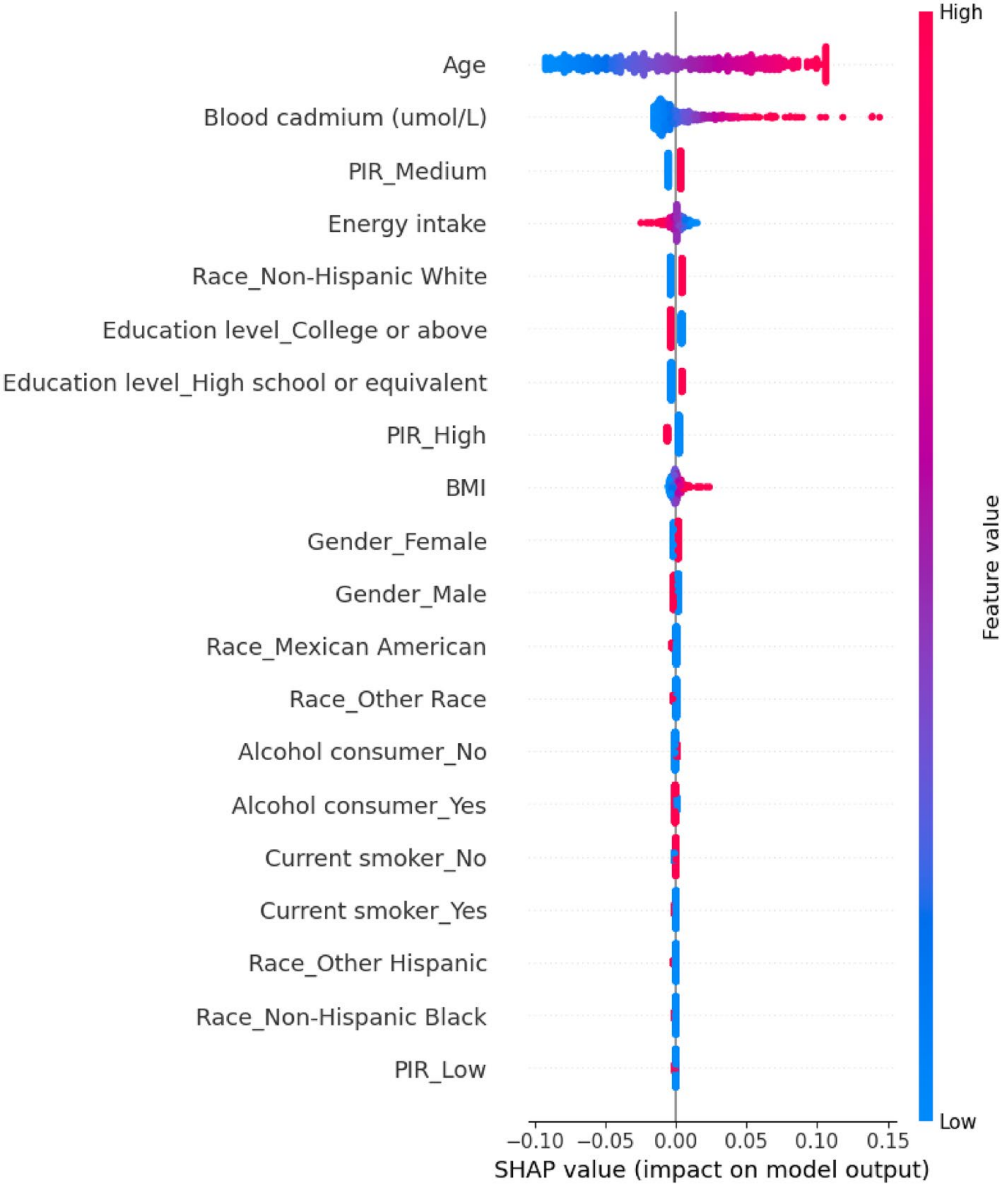
The SHAP summary plot. Shapley values for LR models: each point on the summary plot is the Shapley value for one variable and one instance. All variables are in descending order of importance. The colors represent the values of the variable from low (blue) to high (red). For example, the distribution of characteristics value along the x-axis indicates that low blood cadmium does not help predict stroke risk and high blood cadmium helps predict stroke risk.

The effect of the specified features in the LR model on stroke was depicted graphically using interpretable SHAP values. All variables utilized in the model were represented by Shapley values, as illustrated in Fig. 3. Based on the SHAP values, we observed that blood cadmium made a positive contribution to the model. Furthermore, the figure illustrates that older age, moderate household income, high school or equivalent education, non-Hispanic white ethnicity, high BMI, and energy intake were the most impactful variables in predicting stroke risk.

## Discussion

In this study, we used ML techniques combined with interpretable shap charts to explore the potential predictive ability of blood cadmium for stroke. Among the five ML models considered, the LR model achieved the best performance and was ultimately chosen for stroke identification. By utilizing the SHAP game theory approach, we were able to assess the importance of each selected feature in the model. The LR model showed a comparable level of performance and identified blood cadmium as the key metal contributing significantly to stroke prediction.To date, several studies have reported an association between blood cadmium levels and stroke. Our findings are consistent with previous studies^29,30^. In a cross-sectional study, it was found that a 50% increase in blood cadmium levels corresponded to a 35% increase in the odds of prevalent stroke (OR: 1.35, 95% CI 1.12–1.65) after adjusting for demographic and cardiovascular risk factors^31^. A meta-analytic study involving 4205 strokes revealed a positive association between cadmium exposure and stroke risk, with a relative risk of 1.72 (95% CI 1.29–2.28)^5^. A study from the United States (strong heart study) also showed a non-linear association between urinary cadmium and new-onset stroke, with a hazard ratio of 1.87 (95% CI 1.22–2.86) in Q4 and no significant increase in risk in Q2 or Q3^10^. Additionally, based on the NHANES database, individuals with higher concentrations of cadmium biomarkers exhibited an increased prevalence of a composite measure of myocardial infarction and/or stroke. This association remained consistent even after stratifying by smoking status. The corrected prevalence ratios were 1.54 (95% CI 1.09–2.18) for smokers, 1.57 (95% CI 1.11–2.23) for never-smokers, and 1.31 (95% CI 0.96–1.78) for former smokers^32^.

The heavy metal cadmium, recognized as an environmental pollutant, has been implicated in atherosclerosis, with studies demonstrating that environmental pollution plays a significant role in the development of this condition^33,34^.Cadmium has been associated with several mechanisms that contribute to vascular damage and atherosclerosis^35^.These mechanisms include the formation of reactive oxygen species, promotion of lipid peroxidation, depletion of glutathione (GSH), disruption of sulfhydryl homeostasis, and down-regulation of nitric oxide^36^.

In previous studies, the utilization of machine learning techniques has facilitated the development of predictive models for various adverse health outcomes associated with metal exposure^28,37,38^. However, studies on the construction of predictive models to establish associations between metal exposure and stroke risk are still relatively limited. To the best of our knowledge, this is the first study to develop a predictive model for blood cadmium and stroke risk using ML. We used five ML algorithms to estimate stroke risk for 2664 participants in the NHANES data. Subsequently, we evaluated the ROC curves and AUC values for all models based on the reasonableness of the data.

The results indicated that the LR model outperformed the others, achieving an AUC value of 0.800. Subsequently, we utilized 20% of the primary data for prediction and compared the predicted values with the actual ones, constructing a confusion matrix. The negative prediction value was 0.966, and the overall model accuracy reached 0.966. These findings suggested that blood cadmium can predict stroke to some extent. Hence, the simpler LR model surpassed the more complex models described earlier. However, despite the LR model's high accuracy and AUC, we observed its lower sensitivity due to the limited number of stroke patients in the dataset, leading to a reduced F1 score. To provide a more comprehensive evaluation of the performance of the model, we calculated an average F1 score, which offers a fairer assessment^21^.

Our study also has some limitations. Firstly, we did not incorporate clinical data regarding diseases that may influence stroke, potentially impacting the accuracy of our predictions. Secondly, the diagnosis of stroke relied partially on self-reported information obtained from participants during the US NHANES interview questionnaire, which might have introduced information bias due to cognitive deficits or recall bias. Additionally, due to data constraints, the subtypes and staging of stroke remain unclear. Lastly, despite t the satisfactory performance of the LR model, further external validation is required to ascertain its clinical utility and generalizability.

## Conclusion

We compared the ability of five ML models (KNN, DT, LR, MLP, RF) in predicting stroke with blood cadmium using a database based on the NHANES population. Among these models, the LR model demonstrated superior efficiency, accuracy, and robustness in detecting the relationship between blood cadmium and the risk of stroke. Our findings indicated a significant positive association between elevated blood cadmium levels and the risk of stroke among participants in the NHANES 2013–2014. However, further studies are necessary to validate and confirm our results.

### Supplementary Information

Supplementary Table S1.

## Data Availability

The data in our study are publicly available online from the NHANES (https://www.cdc.gov/nchs/nhanes/index.htm).
