# Author Correction: A machine learning model predicts stroke associated with blood cadmium level

**DOI:** 10.1038/s41598-024-69583-1

**Published:** 2024-08-09

**Authors:** Wenwei Zuo, Xuelian Yang

**Affiliations:** 1 School of Gongli Hospital Medical Technology, University of Shanghai for Science and Technology, No. 516, Jungong Road, Yangpu Area, Shanghai, 200093 China; 2 Department of Neurology, Shanghai Pudong New Area Gongli Hospital, No. 219 Miaopu Road, Pudong New Area, Shanghai, 200135 China

Correction to: *Scientific Repots* 10.1038/s41598-024-65633-w, published online 26 June 2024

In the original version of this Article Xuelian Yang was incorrectly affiliated with ‘School of Gongli Hospital Medical Technology, University of Shanghai for Science and Technology, No. 516, Jungong Road, Yangpu Area, Shanghai, 200093, China’. The correct affiliation is listed below:

Department of Neurology, Shanghai Pudong New Area Gongli Hospital, No. 219 Miaopu Road, Pudong New Area, Shanghai, 200135, China

The original Article has been corrected.

