# Supplementary material for: A machine learning model predicts stroke associated with blood cadmium level
Source: Sci Rep. 2024 Jun 26;14:14739. doi: 10.1038/s41598-024-65633-w (PMC11208606; doi:10.1038/s41598-024-65633-w)
Supplement: Supplementary file 1 — Supplementary Table S1. [file 41598_2024_65633_MOESM1_ESM.docx]

**Table S1** Details of hyperparameter for each machine learning model.

| Model | Hyperparameter |
| --- | --- |
| KNN | algorithm= 'auto', leaf_size= 10, metric= 'manhattan', n_neighbors= 7, weights= 'distance' |
| DT | criterion= 'entropy', max_depth= 5 |
| LR | C= 0.001, penalty= l2 |
| MLP | activation='relu', alpha= 0.0001, hidden_layer_sizes= (50,50), learning_rate= 'constant', solver= 'adam' |
| RF | max_depth: None, max_features: 'auto', min_samples_leaf: 1, min_samples_split: 2, n_estimators: 50 |

KNN, K-Nearest Neighbour; DT, decision tree; LR, logistic regression; MLP, multilayer perceptron; RF, random forest; RF, random forest.
